# Supplementary material for: Message framing materials applied to healthy eating decision-making for pregnant women with gestational diabetes mellitus: An exploratory study
Source: PLoS One. 2025 Feb 24;20(2):e0319416. doi: 10.1371/journal.pone.0319416 (PMC11849819; doi:10.1371/journal.pone.0319416)
Supplement: S1 Appendix — (PDF) [file pone.0319416.s001.pdf]

Table 1 The content and scoring of the material entries in the first rounds of Delphi surveys

Items	content		Expert 1	Expert 2	Expert 3	Expert 4	Expert 5	Expert 6	Expert 7	Expert 8	Expert 9	Expert 10	Expert 11	Expert 12	Expert 13	Expert 14	Expert 15	Expert 16	Expert 17	x≥3.5	s	CV < 0.25
	Positive frame																					
	Immediate effects																					
Item 1	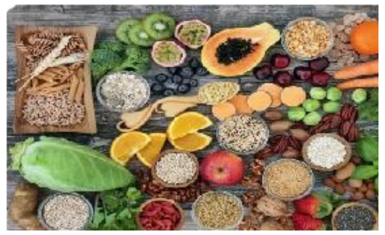	If you consume 2 to 3 fistfuls of carbohydrates, such as grains, beans, and root vegetables, every day, then it may reduce your risk of ketosis and benefit your baby's brain and intellectual development.	5	5	5	5	5	5	5	5	4	5	5	5	5	4	5	5	5	4.88	0.33	0.07
Item 2	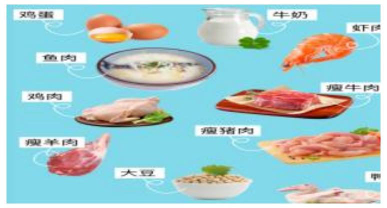	If you consume a moderate amount of fish, shrimp, shellfish, poultry, and other meats, then it may help stabilize your blood sugar and blood lipids, promote your baby's growth and development, and improve your baby's immunity.	5	5	5	5	5	5	5	5	5	5	5	5	5	4	5	5	5	4.94	0.24	0.05
Item 3	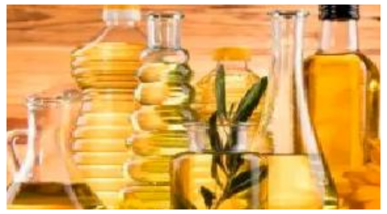	If you consume 1 thumbtip-sized amount of olive oil, tea seed oil, flaxseed oil, and other oils every day, then you may avoid insulin resistance and elevated blood lipids, which is good for blood sugar and weight control and promotes your baby's brain and vision development.	5	5	5	5	4	4	5	5	5	5	5	5	5	4	4	5	4	4.71	0.47	0.10
Item 4	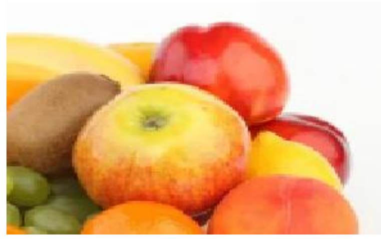	If you consume fistfuls of low-sugar fruits such as apples, peaches, and kiwis every day while your blood sugar is under control, then you may avoid a rapid rise in blood glucose and rapid weight gain and reduce the risk of preterm labor, miscarriage, and other occurrences of your baby.	5	5	5	5	5	5	5	5	4	5	4	5	5	4	1	5	4	4.53	1.01	0.22
Item 5	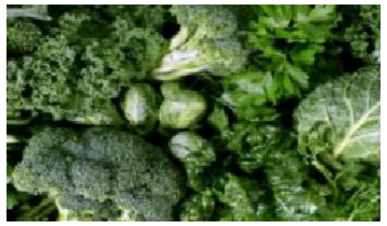	If you consume 1 to 2 large handfuls of spinach, celery, and other green leafy vegetables every day, then it may prevent you from constipation, provide micronutrients, stabilize blood sugar, promote healthy growth, and reduce the risk of developmental stagnation in your baby.	5	5	5	5	5	5	5	5	5	5	5	5	5	4	4	5	5	4.88	0.33	0.07
Item 6	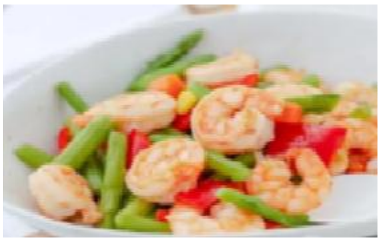	If you often choose to steam, stir-fry or boil your cooking, then it may benefit your blood sugar and weight control.	5	5	5	5	5	5	5	5	5	5	5	5	5	5	3	5	4	4.82	0.53	0.11

**Item 7**	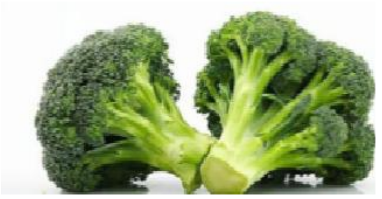	If you often choose non-fermented and chunky foods, then it may help your blood sugar control and make you less likely to feel hungry.	4	5	5	5	4	4	5	4	4	4	5	5	5	4	3	5	4	4.41	0.62	0.14
	**Long-term effects**																					
**Item 8**	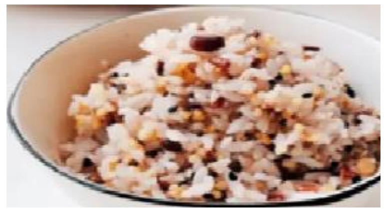	If you consume the right amount of roughage and staple foods, then you may reduce your baby's risk of developing diabetes as he or she grows up.	5	5	5	5	4	5	4	4	5	5	5	5	5	4	3	5	3	4.53	0.72	0.16
**Item 9**	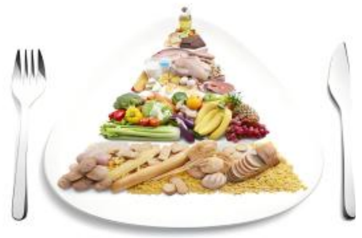	If you make sure you eat enough carbohydrates, then you may reduce the risk of producing too many ketones, which can lead to reduced cognitive and motor function later in life.	4	5	5	5	4	4	4	4	4	5	4	5	5	4	4	5	5	4.47	0.51	0.12
**Item 10**	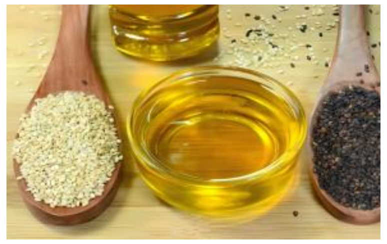	If you consume thumbtip-sized amounts of olive oil, tea seed oil, flaxseed oil, and other oils every day, you may lower your blood cholesterol levels and reduce your risk of cardiovascular disease and obesity later in life.	5	5	5	4	4	3	5	5	5	5	5	5	5	4	3	4	3	4.41	0.80	0.18
**Item 11**	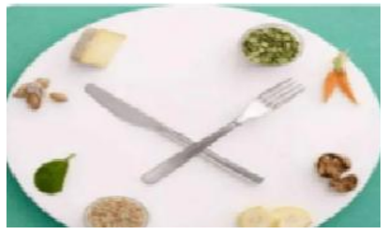	If you choose to eat regular meals with fixed meal times and totals, eat smaller meals, and follow a vegetable-meat-staple food sequence, then it may reduce your risk of developing type 2 diabetes later in life.	4	5	5	4	4	5	4	4	5	5	5	5	5	3	3	5	5	4.47	0.72	0.16
	**negative frame**																					
	**Immediate effects**																					
**Item 12**	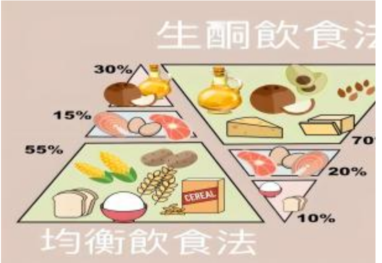	If you consume less than 2–3 fistfuls of carbohydrates per day, such as grains, beans, and root vegetables, then it may increase your risk of ketosis(a disorder of glucose-lipid metabolism), which affects your baby's brain development.	5	5	5	4	5	5	5	5	5	4	5	5	5	4	4	3	5	4.65	0.61	0.13
**Item 13**	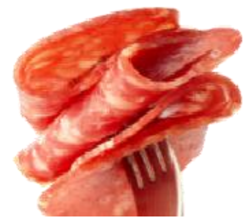	If you consume too much processed meat, such as bacon and tenderloin, then it may not be conducive to the stability of your blood sugar and blood lipids, increase the risk of deformities in your baby, and lower your baby's immunity.	5	5	5	5	4	4	5	4	4	5	4	5	5	4	4	4	4	4.47	0.51	0.12

**Item 14**	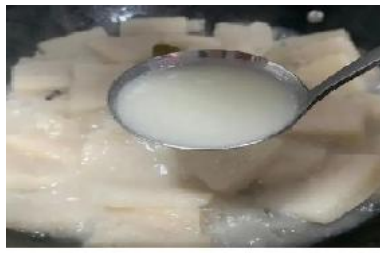	If you consume more than 1 thumbtip-sized oil or choose oils such as lard or butter every day, it may lead to insulin resistance and elevated blood lipids, which is not conducive to blood glucose and weight control and may affect your baby's brain and vision development.	5	5	5	5	5	5	5	4	4	5	4	4	5	3	4	5	4	4.53	0.62	0.14
**Item 15**	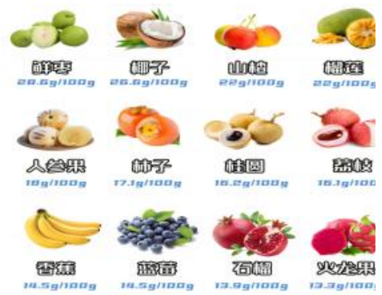	If you consume fist-sized high-sugar fruits such as lychee, longan, and pineapple nectar every day while your blood sugar control is stable, then it may lead to a rapid rise in blood sugar and rapid weight gain, increasing the risk of your baby's preterm labor and miscarriage.	5	5	5	5	5	5	5	3	4	4	5	5	5	2	3	4	5	4.41	0.94	0.21
**Item 16**	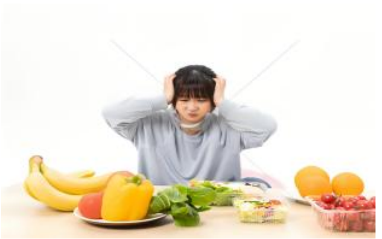	If you consume less than 1 handful of green leafy vegetables per day, such as spinach and celery, then it may lead to constipation, micronutrient deficiencies, and blood sugar fluctuations, which may cause your baby's growth to be slowed down and increase the risk of developmental stagnation.	4	5	5	5	5	5	4	3	4	5	5	4	5	2	2	5	4	4.24	1.03	0.24
**Item 17**	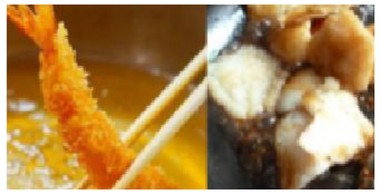	If you often choose to fry, thicken, or deep-fry your cooking, then it may lead to fluctuations in your blood sugar and weight gain.	5	5	5	5	5	5	5	5	5	5	5	4	5	4	4	5	4	4.76	0.44	0.09
**Item 18**	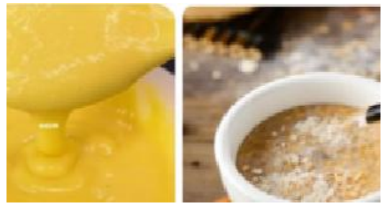	If you often choose food in the form of paste or porridge, then it may lead to a rapid rise in blood sugar and hunger pangs.	5	5	5	5	5	5	5	4	5	4	5	5	5	4	4	5	4	4.71	0.47	0.10
		**Long-term effects**																				
**Item 19**	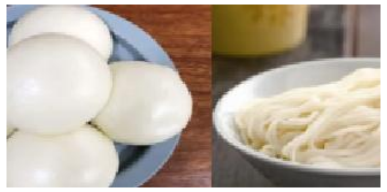	If you consume too many fine white flour staples, then you may increase your baby's risk of developing diabetes when he or she grows up.	4	5	5	5	4	4	5	4	4	4	5	4	5	2	3	5	4	4.24	0.83	0.20
**Item 20**	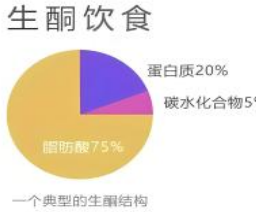	If you overrestrict your carbohydrate intake, then you may increase the risk of producing too many ketones, which can lead to reduced cognitive and motor function later in life.	5	5	5	3	4	4	5	4	5	5	5	5	5	4	4	5	4	4.53	0.62	0.14

**Item 21**	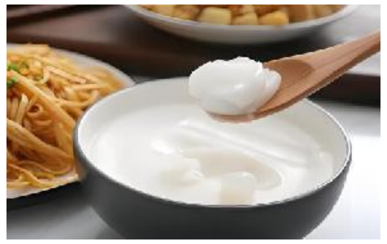	If you consume more than the tip of your thumb or choose oils such as lard or butter, then it may raise your blood cholesterol level and increase your risk of cardiovascular disease and obesity later in life.	4	5	5	5	4	4	5	4	4	5	4	5	5	4	3	4	4	4.35	0.61	0.14
**Item 22**	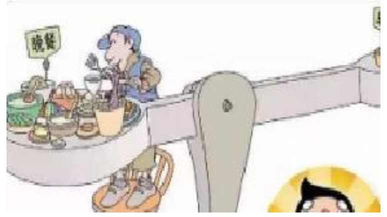	If you choose to eat irregularly, such as irregular meal times and total amount of meals, eating more than one meal and less than one meal, and not following the order of vegetables-meat-staple food, then you may increase your risk of developing type 2 diabetes.	5	5	5	4	5	5	5	4	4	5	4	4	5	2	3	4	4	4.29	0.85	0.20

Table 2 The content and scoring of the material entries in the second rounds of Delphi surveys

Items	content<br>Positive frame<br>Immediate effects		Expert 1	Expert 2	Expert 3	Expert 4	Expert 5	Expert 6	Expert 7	Expert 8	Expert 9	Expert 10	Expert 11	Expert 12	Expert 13	Expert 14	Expert 15	Expert 16	Expert 17	x≥3.5	s	CV < 0.25
**Item 1**	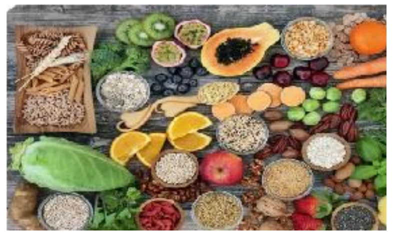	If you consume 2 to 3 fistfuls of carbohydrates, such as grains, beans, and root vegetables, every day, then it may reduce your risk of ketosis (a disorder of glucose-lipid metabolism) and benefit your baby's brain and intellectual development.	5	5	5	5	5	5	5	5	5	5	4	5	5	5	5	5	5	4.94	0.24	0.05
**Item 2**	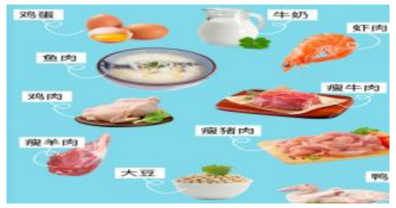	If you consume two palm-sized meats of fish, shrimp, shellfish, poultry, and other meats with a thickness equivalent to the thickness of your little finger every day, then it may help stabilize your blood sugar and blood lipids, promote your baby's growth and development, and improve your baby's immunity.	5	5	5	5	5	5	5	4	5	5	5	5	5	5	5	5	5	4.94	0.24	0.05
**Item 3**	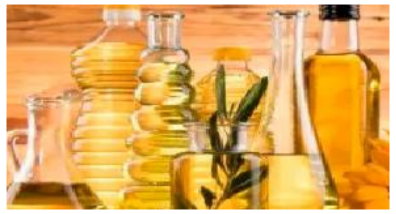	If you consume 1 thumbtip-sized amount of olive oil, tea seed oil, flaxseed oil, and other oils every day, then you may avoid insulin resistance and elevated blood lipids, which is good for blood sugar and weight control and promotes your baby's brain and vision development.	5	5	5	5	5	5	5	5	5	5	5	4	5	5	5	5	5	4.94	0.24	0.05
**Item 4**	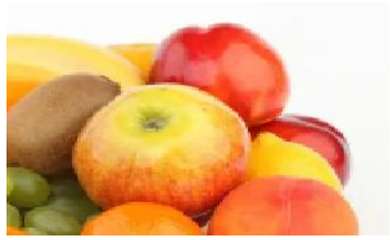	If you choose fruits, a daily intake of 1 fist-sized low-sugar fruit such as apples, peaches, kiwis, etc., then you may avoid a rapid rise in blood glucose and rapid weight gain and reduce the risk of preterm labor, miscarriage, and other occurrences of your baby.	5	5	5	5	5	5	5	5	4	5	4	5	5	5	5	5	5	4.88	0.33	0.07

**Item 5**	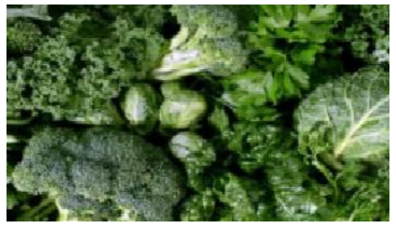	If you consume 1 to 2 large handfuls of spinach, celery, and other green leafy vegetables every day, then it may prevent you from constipation, provide micronutrients, stabilize blood sugar, promote healthy growth, and reduce the risk of developmental stagnation in your baby.	5	5	5	5	5	5	5	5	5	5	5	5	5	5	4	5	5	4.94	0.24	0.05
**Item 6**	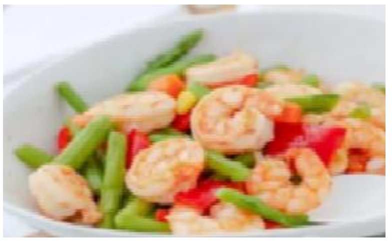	If you often choose to steam, stir-fry, or boil your cooking, then it may benefit your blood sugar and weight control.	5	5	5	5	5	5	5	5	5	5	5	4	5	5	5	5	5	4.94	0.24	0.05
**Item 7**	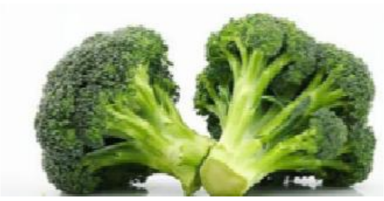	If you often choose unprocessed, chunky foods, then it may help your blood sugar control and make you less likely to feel hungry.	4	5	5	5	4	4	5	4	4	4	4	4	5	5	3	5	4	4.35	0.61	0.14
		**Long-term effects**																				
**Item 8**	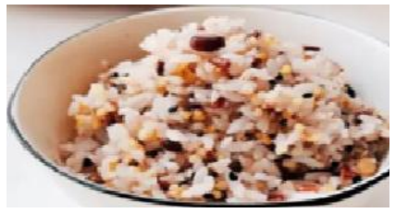	If you consume the right amount of roughage and staple foods, then you may reduce your baby's risk of developing diabetes as he or she grows up.	5	5	5	5	5	5	4	5	5	5	5	5	5	5	5	5	5	4.94	0.24	0.05
**Item 9**	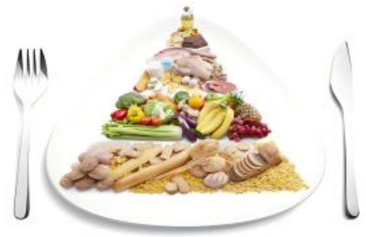	If you make sure you eat enough carbohydrates, then you may reduce the risk of producing too many ketones, which can lead to reduced cognitive and motor function later in life.	4	5	5	5	4	4	4	4	4	4	4	5	5	3	4	5	5	4.35	0.61	0.14
**Item 10**	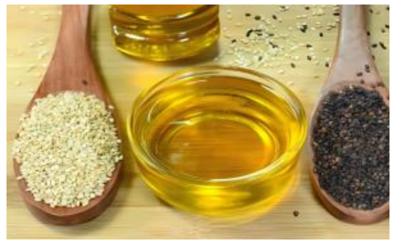	If you consume thumbtip-sized amounts of olive oil, tea seed oil, flaxseed oil, and other oils every day, you may lower your blood cholesterol levels and reduce your risk of cardiovascular disease and obesity later in life.	5	5	5	4	4	5	5	5	5	5	5	5	5	5	5	5	5	4.88	0.33	0.07
**Item 11**	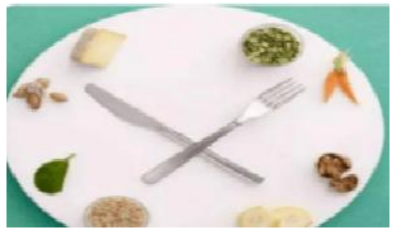	If you choose to eat regular meals with fixed meal times and totals, eat smaller meals, and follow a vegetable-meat-staple food sequence, then it may reduce your risk of developing type 2 diabetes later in life.	4	5	5	5	4	5	4	4	5	5	4	5	5	5	3	5	4	4.53	0.62	0.14

**negative frame**																						
		**Immediate effects**																				
**Item 12**	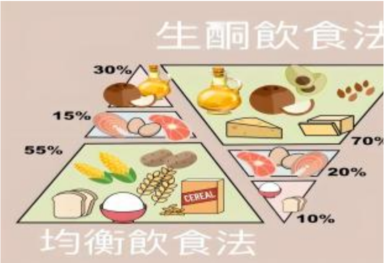	If you consume less than 2–3 fistfuls of carbohydrates per day, such as grains, beans, and root vegetables, then it may increase your risk of ketosis (a disorder of glucose-lipid metabolism), which affects your baby's brain development.	5	5	5	4	5	5	5	5	5	4	5	5	5	5	4	5	5	4.82	0.39	0.08
**Item 13**	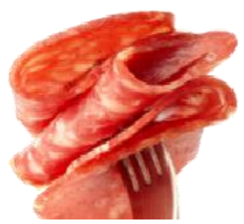	If you consume too much red meat with high fat content, such as pork and rabbit, or processed meat, such as bacon and tenderloin, then it may not be conducive to the stability of your blood sugar and blood lipids, increase the risk of deformities in your baby, and lower your baby's immunity.	5	5	5	5	4	4	5	4	4	5	4	5	5	4	5	4	3	4.47	0.62	0.14
**Item 14**	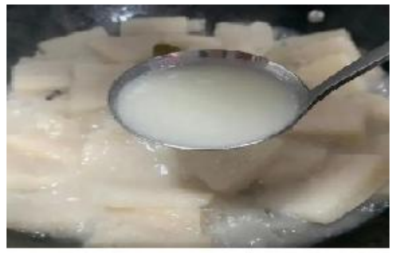	If you consume more than 1 thumbtip-sized oil or choose oils such as lard or butter every day, it may lead to insulin resistance and elevated blood lipids, which is not conducive to blood glucose and weight control and may affect your baby's brain and vision development.	5	5	5	4	5	5	5	4	4	5	4	4	5	4	4	5	4	4.53	0.51	0.11
**Item 15**	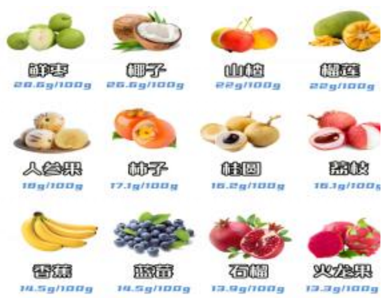	If you choose fruits, you consume too many high-sugar fruits such as lychee, longan, and pineapple nectar every day, then it may lead to a rapid rise in blood sugar and weight gain within a short period of time, which may increase the risk of your baby's preterm labor and miscarriage.	5	5	5	5	5	5	5	5	5	4	5	5	5	5	5	4	5	4.88	0.33	0.07
**Item 16**	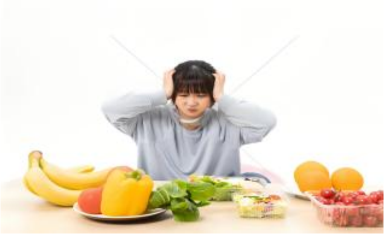	If you consume less than 1 handful of green leafy vegetables per day, such as spinach and celery, then it may lead to constipation, micronutrient deficiencies, and blood sugar fluctuations, which may cause your baby's growth to be slowed down and increase the risk of developmental stagnation.	4	5	5	5	5	5	4	5	5	5	5	5	5	5	4	5	5	4.82	0.39	0.08

**Item 17**	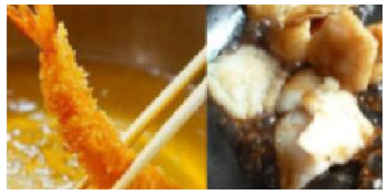	If you often choose to fry, thicken, or deep-fry your cooking, then it may lead to fluctuations in your blood sugar and weight gain.	5	5	5	5	5	5	4	5	5	5	4	5	5	5	5	5	4	4.82	0.39	0.08
**Item 18**	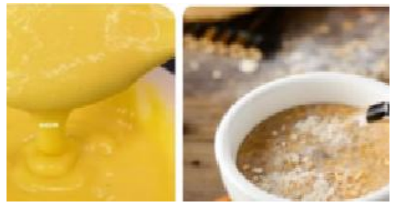	If you often choose processed, porridge-like foods, then it may lead to a rapid rise in blood sugar and hunger pangs.	5	5	5	5	5	5	5	4	5	5	5	5	5	5	5	4	5	4.88	0.33	0.07
	**Long-term effects**																					
**Item 19**	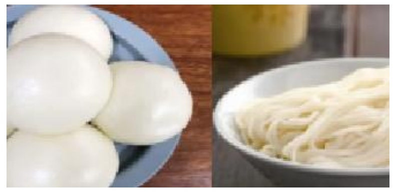	If you consume too many fine white flour staples, then you may increase your baby's risk of developing diabetes when he or she grows up.	4	5	5	4	4	4	5	4	5	4	5	4	5	3	4	5	4	4.35	0.61	0.14
**Item 20**	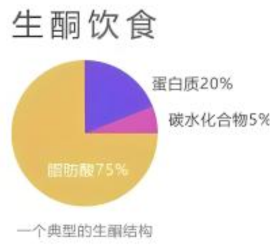	If you overrestrict your carbohydrate intake, then you may increase the risk of producing too many ketones, which can lead to reduced cognitive and motor function later in life.	5	5	5	5	4	4	5	4	5	5	4	4	5	5	3	5	5	4.59	0.62	0.13
**Item 21**	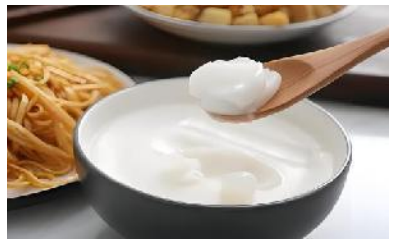	If you consume more than the tip of your thumb or choose oils such as lard or butter, then it may raise your blood cholesterol level and increase your risk of cardiovascular disease and obesity later in life.	5	5	4	4	5	4	4	5	4	4	4	5	4	5	4	4	3	4.29	0.59	0.14
**Item 22**	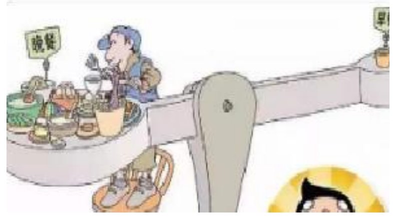	If you choose to eat irregularly with irregular meal times and totals, overeating, eating too fast, and irregular meals such as soup and rice, then you may increase your risk of developing type 2 diabetes.	5	5	5	4	5	5	5	4	4	5	4	4	5	3	4	4	4	4.41	0.62	0.14
